# Extrinsic tooth staining potential of high dose and sustained release iron syrups on primary teeth

**DOI:** 10.1186/s12903-015-0072-0

**Published:** 2015-08-04

**Authors:** Sharat Chandra Pani, Fahad Murdhi Alenazi, Abdullah Muhammad Alotain, Hamad Daher Alanazi, Abdullah Saeed Alasmari

**Affiliations:** Pediatric and Preventive Dentistry, Riyadh Colleges of Dentistry and Pharmacy, Riyadh, Saudi Arabia; Riyadh Colleges of Dentistry and Pharmacy, Riyadh, Saudi Arabia; Department of Preventive Dental Sciences, PO Box 84891, Riyadh, 11681 Kingdom of Saudi Arabia

**Keywords:** Oral iron supplement, Ferrous fumarate, Ferric oxide polymaltose, Staining of teeth, Primary teeth

## Abstract

**Background:**

Iron in the form of oral supplements is routinely prescribed to children to help fight anemia, however tooth staining is a commonly reported complication. This study tests in vitro, the staining potential of two different forms of iron syrup on primary teeth.

**Methods:**

Forty caries free primary central incisors were divided into four groups of ten teeth each. The control group comprised of ten teeth immersed in artificial saliva, while the test solutions were comprised of different forms of iron mixed with vitamins such that the iron content of each solution was approximately 100 mg (from 100 to 101.1 mg). The test solutions used iron syrup (Ferrose®, SPIMACO, Jeddah, Saudi Arabia) with iron in the form of ferric oxide polymaltose (FOP), slow release formula (Ferroglobin®, Vitabiotics ltd., London, UK) containing ferrous fumarate (FF and a combination of the two (FOP + FF). All the teeth were then immersed for 72 h and subjected to a protocol developed by Lee et al. to test staining. Color changes were measured using a wave dispersion spectro-photometer (Color-Eye 7000A, X-Rite Gmbh, Regensdorf, Switzerland) on the exposed labial surface at 4, 8, 24, 48 and 72 h. Two-way ANOVA with Scheffe’s post hoc test was used to determine significance of difference in shade, while the Kurskull-Wallis test used to determine the significance of difference in clinical staining (∆E > 3).

**Results:**

While all three iron groups showed some amount of staining, the combination of the two forms of iron (FOP+FF) showed significantly lower incidence of clinical staining than the other two groups at the end of 72 h. At the end of 72 h the (FOP) had significantly higher ∆E than ferrrous fumarate (FF ) while the combination (FOP+ FF) had a significantly lower ∆E than either group.

**Conclusion:**

In an in vitro model, combining different forms of iron seems to elicit a lower intensity of staining than equivalent doses of a single form of iron.

**Electronic supplementary material:**

The online version of this article (doi:10.1186/s12903-015-0072-0) contains supplementary material, which is available to authorized users.

## Background

Iron is found in all living tissues including enamel, dentin and the dental pulp [1, 2]. Iron deficiency affects more than two billion people in the world and is one of the most common nutritional deficiencies [3]. Although iron deficiency may sometimes result from defects in the body’s ability to metabolize iron, the most common cause is inadequate iron in the diet, a deficiency that is treatable by foods and supplements which contain iron salts [3, 4]. Iron salts in the form of supplements, usually drops or syrups fortified with folic acid and/or vit. B12, are often prescribed to children below 5 years of age [3]. While drops and syrups containing iron, folic acid and Vit B12 have traditionally been the standard form of dispensing iron salts, companies also incorporate these ingredients into infant formulas [4], and multi-vitamin formulas [5].

The extrinsic staining of primary teeth is often a cause of concern to parents and there have been reports that such staining can adversely affect the social interactions of preschool children [6]. Since ferric sulfide is the chemical insoluble form of iron supplement, it has been suggested that it may interact (in ionic form) with gingival cervical fluid and the bacterial hydrogen sulfide to produce iron stains [7]. There is ample documentation of iron staining reported on the teeth of children taking iron syrups, drops and other preparations [5, 8–10].

While it has been shown that low dose iron in the form of ferrous fumarate produces less tooth staining than syrups [5], there is concern that these low dose formulas do not provide adequate iron to prevent anemia [11]. This has meant that physicians often prescribe high dose iron syrups, with iron in the form of ferrous sulfate, or more recently ferric hydroxide polymaltose complex to help combat anemia [12]. There is little evidence to highlight the staining potential of either the low dose ferrous fumarate preparations or the newer ferric hydroxide polymaltose complex on primary teeth.

There are several methods to evaluate the change of tooth color due to the deposition of stains, wavelength dispersive spectro-photometry has been shown to be the most accurate method for the assessment of dental stains in general [13]. Furthermore the assessment of color changes in the tooth in vitro has been shown to yield reproducible results when done using a standardized in vitro staining model [14].

The aim of the present study was to compare the staining potential of iron supplements in the form of a ferric hydroxide polymaltose and vitamin iron syrup, ferrous fumarate iron and vitamin formula and a combination of the two, using an in vitro staining model.

## Methods

The current study used an in vitro experimental model. Institutional ethical consent was obtained from the ethics committee of the Riyadh Colleges of Dentistry and Pharmacy and project was assigned the number USRP/2013/76.

### Teeth used

Forty caries free primary incisors extracted in patients where parents’ desired extraction due to pre-shedding mobility were used in the study. Teeth selected were free from developmental defects, enamel hypoplasia, restorations or existing extrinsic or intrinsic stains. The parents were informed about the use of the teeth for research purposes and written informed consent was obtained from the parents. All teeth used in this study were extracted in a one month period from patients reporting to the dental clinics of the college and sterilized by immersing in 10 % formalin for 24 h by the clinical staff before the teeth were made available for research purposes. Once received, the teeth were thoroughly rinsed and then stored in artificial saliva (Caphosol® Cytogen Corp. Princeton NJ, USA ) from the time of extraction until usage. The teeth were rinsed thoroughly and then embedded in a circular acrylic resin mold 4 cm in diameter such that the labial surface of these incisors was visible through a 2cm x 2cm window. The teeth were then randomly divided into four groups of ten teeth each and each group was immersed in one of the four prepared solutions.

### Preparation of solutions

Control Solution −250 ml of artificial saliva (Caphosol® Cytogen Corp.).

Ferric Oxide Polymaltose Group (FOP) – 10 ml (maximum therapeutic daily dose) containing of commercially available iron, folic acid and vitamin B12 syrup (Ferrose®, SPIMACO, Jeddah, Saudi Arabia), was diluted with artificial saliva (Caphosol® Cytogen Corp.) to make up 250 ml such that the total available iron content in the solution was 100 mg in the form of ferric hydroxide polymaltose complex.

Ferrous Fumarate Group (FF) – 35 ml of slow release formula (Ferroglobin®, Vitabiotics ltd., London, UK) was diluted with artificial saliva (Caphosol® Cytogen Corp.) to make up 250 ml such that the total available iron content in the solution was approximately 100 mg (100.2 mg) in the form of ferrous fumarate.

Combination solution (FOP + FF ) – 10 ml of iron, folic acid and vitamin B12 syrup (Ferrose®, SPIMACO, Jeddah, Saudi Arabia) was combined with 15 ml of slow release formula (Ferroglobin®, Vitabiotics ltd., London, UK ) to produce 35 ml of a high concentration iron syrup (total iron 142.94 mg). Twenty five milliliters of this was diluted with artificial saliva (Caphosol® Cytogen Corp.) to make up 250 ml such that the total available iron content in the solution was approximately 100 mg (101.1mg).

### Shade assessment

Baseline shades of all teeth were measured using a wave dispersion spectro-photometer (Color-Eye 7000A, X-Rite Gmbh, Regensdorf, Switzerland). The samples were removed from the solution at 4, 8, 4, 48 and 72 h and rinsed with distilled water. Shade was measured on the exposed labial surface using the protocol developed by Lee et al. [14] to obtain the CIE l*a*b values for change in color. The change in color ΔE was calculated using the formula $\Delta {E}_{\mathrm{ab}}=\sqrt{{\left({L}_2^{\ast }-{L}_1^{\ast}\right)}^2+{\left({a}_2^{*}-{a}_1^{*}\right)}^2+{\left({b}_2^{*}-{b}_1^{*}\right)}^2}$ A ∆E >3 (from the baseline score) was recorded as clinically visible staining.

### Statistical analyses

The baseline l* a* b values of the teeth assigned to each group were compared using the One-Way ANOVA to test the effectiveness of randomization. The ΔE values across groups were then subjected to statistical analysis using the repeated measures two-way ANOVA and the Scheffe’s post-hoc test to compare the intensity of staining between groups. The total number of teeth exhibiting clinically visible staining was tabulated and was tested for statistical significance using the Kurskull-Wallis test and Mann–Whitney –U between different groups as a post-hoc test. All analysis was done using the SPSS ver. Twenty two data processing software. The level of significance was set at *p* < 0.05. Since multiple comparisons with the Mann–Whitney-U were performed a Bonferroni correction was applied and the level of significance was set at *p* < 0.01.

## Results

There was no significant difference between the mean baseline L*,a*,b* values of the teeth assigned to the different groups (Table 1).

**Table 1:** Mean baseline L*, a*, b* values for teeth assigned to each group

Value	Mean (+/− SD)				Sig*
	Control	FF	FOP	FF + FOP	
L*	80.81 (+/− 1.7)	80.28(+/−2.1)	80.49(+/−2.3)	80.87(+/−2.5)	.921
a*	.278(+/−1.04)	.374(+/−1.02)	.178(+/−1.0)	.243(+/−.96)	.978
b*	15.82(+/−2.6)	16.14(+/−2.6)	17.64(+/−2.9)	16.88(+/−2.4)	.421

* significance calculated using the One-Way ANOVA

**Fig. 1:**
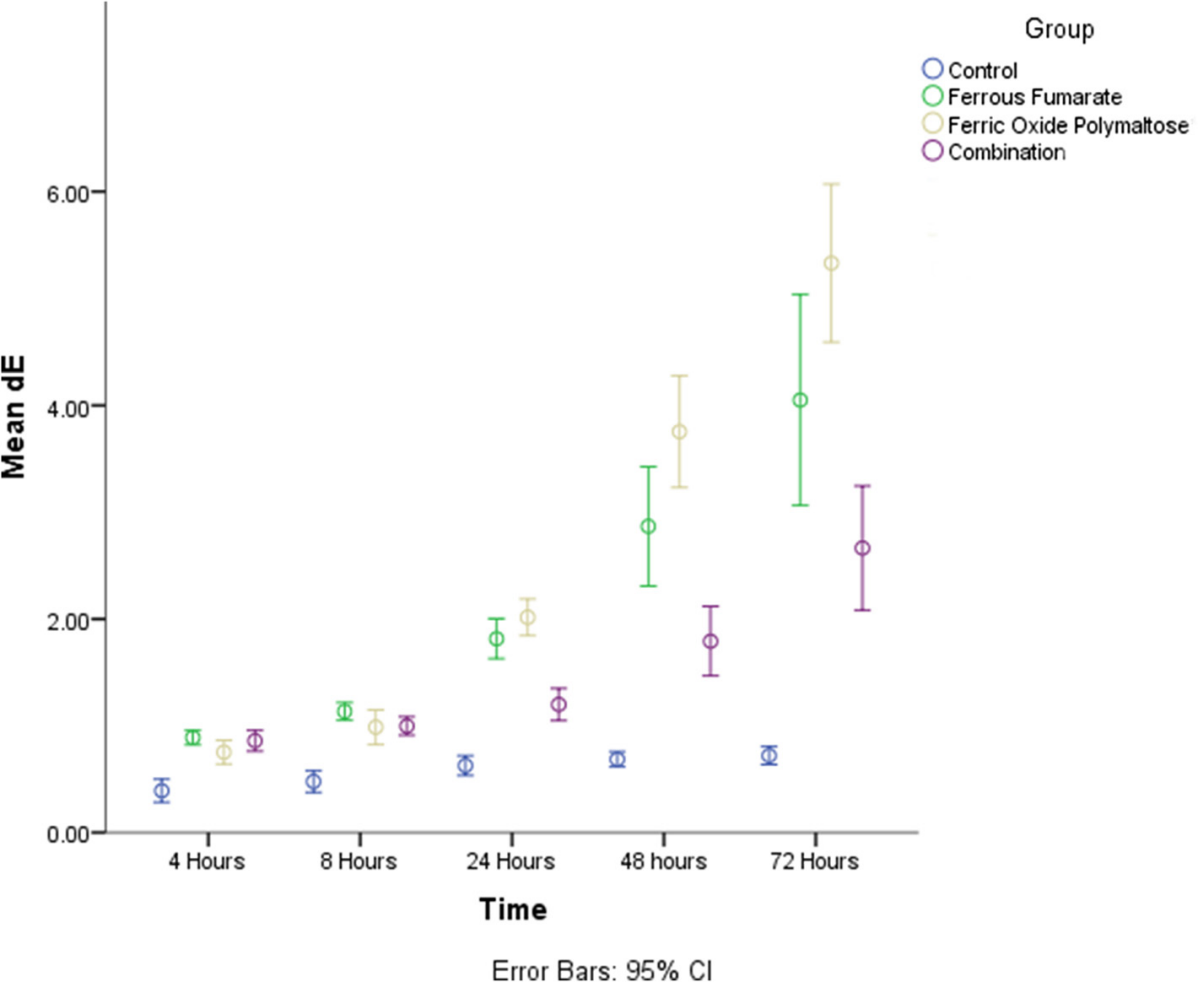
The effect of time of immersion on the ∆E of the different groups of solutions. Difference in alphabet indicates significant difference (*p* < 0.05) between groups at a given time interval while difference in symbol indicates difference between time interval for each group (*p* < 0.05). Significance calculated using the Two Way Repeated Measures ANOVA and Scheffe’s Post-hoc test

When the repeated measures two-way ANOVA was used to check the progression of staining among the different groups (Fig. 1) it was observed that the control group showed no significant increase in ∆E across time intervals. The test revealed significant differences across time intervals (*p* < 0.001) for each of the iron containing groups. Post-hoc tests showed that for the FF group there was no significant difference at the 4, 8 and 24 h interval (*p* = .102) however there was a significant increase in ∆E at the subsequent 48 and 72h readings (*p* < 0.05). For the FOP group there was no significant change in ∆E at the 4 and 8 h interval however there was a significant increase in ∆E at the 24, 48 and 72 h intervals (*p* < 0.05). For the FF + FOP group there was no significant difference in the 4, 8 and 24 h groups (*p* = .614), no significant difference between the 24 and 48 h groups (*p* = .104) and a significant increase at 72 h (*p* < 0.05). The findings are summarized in Fig. 1.

A comparison of mean ∆E values across the different groups at 4 and 8 h showed that while the ∆E of the control group was significantly lower than that of the iron containing groups (*p* < 0.05), there was no significant difference between the FF, FOP and FF + FOP groups. At 24 h the control group had significantly lower ∆E than the iron containing groups, however the ∆E of the FF + FOP was significantly (*p* < 0.05) lower than that of the FF and FOP groups. There was no significant difference in the ∆E between the FF and FOP groups (*p* = .252). By 48 h each of the groups had significantly higher ∆E values than control group. The FOP group showed significantly higher ∆ E than all other groups. These findings were also true at 72 h (Fig. 1). There ∆E of the FF + FOP group was significantly lower than the ∆E of the FF group at both 48 and 72 h.

The teeth were examined for clinically visible staining (ΔE >3) only at the end of the72 h cycle. It was observed that none of the teeth in the control group showed any visible staining at the end of 72 h. All ten teeth in the FOP group showed clinical staining, while eight out of ten teeth in the FF group showed clinical signs of staining. Only four out ten teeth in the FOP + FF group showed clinical staining. It was seen that both the FF groups had significantly greater number of stained teeth when compared to the FF + FOP group, while there was no significant difference in the number of stained teeth between the FF and the FOP group or between the FF group and the FF + FOP group(Table 2). Since none of the teeth of the control group showed any clinical staining they were not included in the tests of significance to eliminate unnecessary comparisons or false significant statistics.

**Table 2:** Significance of clinical staining between groups

	Group	Teeth showing clinical staining (N)	Mean rank	Sig^c^
Presence of clinical staining	FF	8^ab^	15.50	0.016
	FOP	10^b^	20.00	
	FF + FOP	4^a^	11.00	

^a,b^Values with different superscripts have a significant difference (*p* < 0.01) when measured using the Mann–Whitney U test

^c^Calculated using the Kurskull Wallis Test

## Discussion

The staining of teeth after administration of oral iron supplements is not a universal phenomenon although some studies have reported incidence of tooth staining in over half the children receiving such supplements [3, 6, 15]. Since the aim of this study was to compare the staining potential of different forms of iron rather than study the actual staining potential of iron, we slected an in-vitro model that was designed to induce staining rather [14] than one of the newer staining models that are designed to mimic cyclic exposure to stains [16]. To this end, we had to follow a standardized protocol developed by Lee et al. [14]; which has been used extensively to study the staining potential of substances with a similar pattern of exposure such as juices, coffees and colas [17, 18].

The ideal dose of additional iron has been a source of debate with literature showing that while iron drops and syrups contain between 12.5 to 50 mg of iron per dose (5 ml), slow release iron formulae contain between 4 to 14.5 mg per dose [5, 12, 19]. While it has been shown that low dose iron in the form of ferrous fumarate produces less tooth staining than syrups [5], there is concern that these low dose formulas do not provide adequate iron to prevent anemia [11]. It was for this reason that this experiment sought to measure staining potential of the supplements such that the iron content of the staining solution would be roughly equal to the prescribed therapeutic dose for a child with iron deficiency anemia (100 mg/day) [3].

The combination of low dose ferrous fumarate with high dose iron supplements has previously been shown to reduce the side effects of iron [20]. Iron in the ferrous form is more rapidly absorbed than in the ferric form and combination regimens often prescribe higher doses of ferric iron than ferrous iron [20–22]. It was for this reason that we decided to combine the ferric oxide polymatose with the ferrous fumarate in a ratio of 2:1. The results of this experiment showed that in equivalent doses, the intensity of stain produced by the solution containing ferrous fumarate is significantly lower than the stain produced by ferric hydroxide polymaltose; however the combination of the two forms of iron produced significantly lower ΔE values than equivalent doses of either.

The results show that while the FOP group started to show significant change in ∆E after 24 h in the model, both the FF and FF + FOP groups required 48 h before the ∆E values started to increase significantly. This could be due to the rapid nature chemical interaction of the ferric form of iron as compared to the ferrous [5]. Nevertheless the fact that significant changes in ∆E only began to emerge after 24 h of continuous exposure may explain why the staining of teeth after the use of iron supplements is not a universally reported phenomenon [3, 5, 9, 12]. The intensity of color change as recorded using the spectrophotometer are reflected by ΔE values (Additional file 1), however it must be remembered that ΔE values <2 are not perceptible to the naked eye. For the purpose of this experiment it was decided to designate a ΔE value of >3 as the presence of clinical staining in keeping with the protocol followed in previous studies [23, 24]. It is interesting to note that the number of stained teeth at the end of 72 h was significantly lower in the group that were immersed in a solution that combined iron in the form of ferrous fumarate with iron in the form of ferric hydroxide polymaltose. While previous research has shown that combining different forms of iron may reduce gastric irritation [21, 22], the results of this study suggest that the hypothesis may also be true for staining of teeth. While our study did not measure the availability of iron in each of the three solutions, previous research has shown that combining different forms of iron does not in any way reduce bio-availability [21, 22].

The results of this study must be viewed keeping in mind its limitations. No preservative was used to prevent possible bacterial growth during storage although mandatory sterilization by immersion in 10 % formalin for 24 h was carried out by the clinical staff in keeping with recommended guidelines [25]. The exact mechanism by which iron causes tooth staining is still not fully clear, with the hypothesis that iron binds to the pellicle being the most accepted theory [6, 9]. The in vitro study model used in this study was one that was designed to induce staining, and it was for this reason that although the teeth were rinsed, they were not polished to remove pellicle. It must be emphasized that in reality the incidence of clinical staining is far lower than that observed in this study. However, it has been shown that exaggerated staining protocols are useful in demonstrating the reduction in staining potential and reduced or absence of staining of teeth in such models can be indicative of low risk of clinical staining [26]. The potential of iron to stain teeth assumes greater significance in the light of recent research into the effect of iron on dental erosion and studies into the feasibility of incorporating iron into mouth-rinses and aerated beverages [27]. The fact that a majority of the teeth placed in the solution that combined ferric and ferrous forms of iron is encouraging and must be viewed keeping in mind sporadic pediatric literature that have shown that combination of these two forms of iron can reduce side effects [12, 20, 28]. While the results of this study do not offer conclusive evidence to frame a hypothesis the topic merits further research.

## Conclusion

The results of this study indicate that iron has staining potential on teeth when tested in vitro and ferrous oxide polymaltose (FOP) presents worse results after longer exposure periods (48 h). Both the occurrence of clinical staining and the intensity of the staining are reduced when the forms of iron are combined in an in vitro model for 24h or more.

## Additional file

Additional file 1: Table S1. Original Δ E values of the samples tested. (XLSX 15 kb)

## References

[1] Eshghi A, Kowsari-Isfahan R, Rezaiefar M, Razavi M, Zeighami S (2012). Effect of iron containing supplements on rats’ dental caries progression. J Dent (Tehran).

[2] Wen X, Paine ML (2013). Iron deposition and ferritin heavy chain (Fth) localization in rodent teeth. BMC Res Notes.

[3] De-Regil LM, Jefferds ME, Sylvetsky AC, Dowswell T (2011). Intermittent iron supplementation for improving nutrition and development in children under 12 years of age. Cochrane Database Syst Rev.

[4] Adcock KG, Hogan SM (2008). Extrinsic iron staining in infant teeth from iron-fortified formula and rice cereal. J Pediatr Pharmacol Ther.

[5] Christofides A, Asante KP, Schauer C, Sharieff W, Owusu-Agyei S, Zlotkin S (2006). Multi-micronutrient Sprinkles including a low dose of iron provided as microencapsulated ferrous fumarate improves haematologic indices in anaemic children: a randomized clinical trial. Matern Child Nutr.

[6] Kumar A, Kumar V, Singh J, Hooda A, Dutta S (2012). Drug-induced discoloration of teeth: an updated review. Clin Pediatr (Phila).

[7] Addy M, Moran J (1995). Mechanisms of stain formation on teeth, in particular associated with metal ions and antiseptics. Adv Dent Res.

[8] Talebi M, Parisay I, Mokhtari N (2012). The parents’ knowledge and behavior towards the effects of using iron supplements on tooth staining and dental caries in Mashhad, Iran. Dent Res J (Isfahan).

[9] Nordbo H, Eriksen HM, Rolla G, Attramadal A, Solheim H (1982). Iron staining of the acquired enamel pellicle after exposure to tannic acid or chlorhexidine: preliminary report. Scand J Dent Res.

[10] Watts A, Addy M (2001). Tooth discolouration and staining: a review of the literature. Br Dent J.

[11] Griffin IJ, Cooke RJ, Reid MM, McCormick KP, Smith JS (1999). Iron nutritional status in preterm infants fed formulas fortified with iron. Arch Dis Child Fetal Neonatal Ed.

[12] Rao R, Georgieff MK (2009). Iron therapy for preterm infants. Clin Perinatol.

[13] Tantbirojn D, Douglas WH, Ko CC, McSwiggen PL (1998). Spatial chemical analysis of dental stain using wavelength dispersive spectrometry. Eur J Oral Sci.

[14] Lee BS, Huang SH, Chiang YC, Chien YS, Mou CY, Lin CP (2008). Development of in vitro tooth staining model and usage of catalysts to elevate the effectiveness of tooth bleaching. Dent Mater.

[15] Sales-Peres SH, Pessan JP, Buzalaf MA (2007). Effect of an iron mouthrinse on enamel and dentine erosion subjected or not to abrasion: an in situ/ex vivo study. Arch Oral Biol.

[16] Ren YF, Feng L, Serban D, Malmstrom HS (2012). Effects of common beverage colorants on color stability of dental composite resins: the utility of a thermocycling stain challenge model in vitro. J Dent.

[17] Baroudi K, Hassan NA (2014). The effect of light-activation sources on tooth bleaching. Niger Med J.

[18] Zanjani VA, Ghasemi A, Torabzadeh H, Jamali M, Razmavar S, Baghban AA (2015). Bleaching effect of ozone on pigmented teeth. Dent Res J (Isfahan).

[19] Quinn EA (2014). Too much of a good thing: evolutionary perspectives on infant formula fortification in the United States and its effects on infant health. Am J Hum Biol.

[20] Liu TC, Lin SF, Chang CS, Yang WC, Chen TP (2004). Comparison of a combination ferrous fumarate product and a polysaccharide iron complex as oral treatments of iron deficiency anemia: a Taiwanese study. Int J Hematol.

[21] Fernandez-Gaxiola AC, De-Regil LM (2011). Intermittent iron supplementation for reducing anaemia and its associated impairments in menstruating women. Cochrane Database Syst Rev.

[22] Pena-Rosas JP, De-Regil LM, Dowswell T, Viteri FE (2012). Intermittent oral iron supplementation during pregnancy. Cochrane Database Syst Rev.

[23] Guler AU, Yilmaz F, Kulunk T, Guler E, Kurt S (2005). Effects of different drinks on stainability of resin composite provisional restorative materials. J Prosthet Dent.

[24] Watanabe H, Kim E, Piskorski NL, Sarsland J, Covey DA, Johnson WW (2013). Mechanical properties and color stability of provisional restoration resins. Am J Dent.

[25] Lee JJ, Nettey-Marbell A, Cook A, Pimenta LA, Leonard R, Ritter AV (2007). Using extracted teeth for research: the effect of storage medium and sterilization on dentin bond strengths. J Am Dent Assoc.

[26] Moreira AD, Mattos CT, de Araujo MV, Ruellas AC, Sant'anna EF (2013). Chromatic analysis of teeth exposed to different mouthrinses. J Dent.

[27] Kato MT, Buzalaf MA (2012). Iron supplementation reduces the erosive potential of a cola drink on enamel and dentin in situ. J Appl Oral Sci.

[28] Grzegorzewska AE (2007). Administration of iron-containing drugs in non-dialyzed patients with chronic kidney disease. Pol Arch Med Wewn.

